# Repercussion of nonsteroidal anti-inflammatory drugs on the gene expression of human osteoblasts

**DOI:** 10.7717/peerj.5415

**Published:** 2018-08-14

**Authors:** Lucia Melguizo-Rodríguez, Víctor J. Costela-Ruiz, Francisco J. Manzano-Moreno, Rebeca Illescas-Montes, Javier Ramos-Torrecillas, Olga García-Martínez, Concepción Ruiz

**Affiliations:** 1 Biomedical Group (BIO277), Department of Nursing, Faculty of Health Sciences, University of Granada, Granada, Spain; 2 Instituto Investigación Biosanitaria, ibs.Granada, University of Granada, Granada, Spain; 3 Biomedical Group (BIO277), Department of Stomatology, School of Dentistry, University of Granada, Granada, Spain; 4 Biomedical Group (BIO277), Department of Nursing, Faculty of Health Sciences, University of Granada, Melilla, Spain; 5 Institute of Neuroscience, University of Granada, Granada, Spain

**Keywords:** Bone tissue, Osteoblasts, Differentiation, Gene expression, NSAIDs

## Abstract

**Background:**

Nonsteroidal anti-inflammatory drugs (NSAIDs) are frequently used in clinical practice, which can have adverse effects on the osteoblast. The objective of this study was to determine the effect of NSAIDs on the osteoblast by analyzing the gene expression of different markers related to osteoblast maturation and function when treated *in vitro* with different NSAIDs.

**Methods:**

Three human osteoblast lines from bone samples of three healthy volunteers were treated with 10 µM acetaminophen, indomethacin, ketoprofen, diclofenac, ibuprofen, ketorolac, naproxen, and piroxicam. The gene expression of different markers (run related transcription factor 2 [*RUNX-2*], type 1 collagen [*COL-I*], osterix [*OSX*], osteocalcin [*OSC*], bone morphogenetic protein 2 [*BMP-2*] and 7 [*BMP-7*], transforming growth factor *β*1 [*TGF-*β*1*], and *TGF*β** receptors [*TGF*β*R1, TGF*β*R2; TGFBR3*]) were analyzed by real-time PCR at 24 h of treatment.

**Results:**

Expression of *RUNX-2, COL-I, OSX*, was reduced by treatment with all studied NSAIDs, *OSC* expression was reduced by all NSAIDs except for ketoprofen, naproxen, or piroxicam. Expression of *BMP-7* was reduced by all NSAIDs; *BMP-2* was reduced by all except for naproxen. In general, NSAID treatment increased the expression of *TGF-*β*1*, but not of its receptors (*TGF*β*-R1, TGF*β*-R2,* and*TFG*β*-R3*), which was either unchanged or reduced by the treatment.

**Conclusion:**

These data confirm that NSAIDs can affect osteoblast physiology, suggesting their possible impact on bone.

## Introduction

Nonsteroidal anti-inflammatory drugs (NSAIDs) comprise a heterogeneous group of drugs, most of which are organic acids with anti-inflammatory, analgesic, antipyretic, and platelet antiaggregant actions. They are frequently used although various adverse gastrointestinal, renal, cardiovascular, or bone effects have been reported. (Danelich et al., 2015; García-Martínez et al., 2015; Harirforoosh, Asghar & Jamali, 2013; Nadanaciva et al., 2013; Scarpignato & Hunt, 2010; Vallano, Llop & Bosch, 2002).

NSAIDs act by inhibiting cyclooxygenase (COX). However, the effect observed on bone tissue does not seem to be exclusively due to COX inhibition. Various studies have demonstrated that NSAIDs can interfere with bone formation and repair by cell cycle arrest in the G0/G1 phase of the osteoblast (Chang et al., 2009; Díaz-Rodríguez et al., 2010; De Luna-Bertos et al., 2015). This may explain the reduced bone density associated with NSAID consumption (Van Staa, Leufkens & Cooper, 2000; Beck et al., 2003; Gerstenfeld et al., 2003; Vuolteenaho, Moilanen & Moilanen, 2008; Pountos et al., 2012) and the *in vitro* growth inhibition shown by osteoblastic cells in the presence of these drugs (Díaz-Rodríguez et al., 2010; De Luna-Bertos et al., 2013; Evans & Butcher, 2004; Díaz-Rodríguez et al., 2012a; Díaz-Rodríguez et al., 2012b; García-Martínez et al., 2011). These drugs also inhibit the maturation/differentiation of this cell population (Díaz-Rodríguez et al., 2012a; Díaz-Rodríguez et al., 2012b; De Luna-Bertos et al., 2013).

Osteoblasts play an essential role in bone physiology, participating in bone formation and remodeling and in the regeneration of damaged bone tissue (Datta et al., 2008; Long, 2011). The maturation and function of this cell population are highly complex processes involving autocrine, paracrine, and endocrine factors (Florencio-Silva et al., 2015). The objective of this study was to analyze the possible effect of NSAIDs on the gene expression of different markers involved in osteoblast maturation/differentiation and function by using an *in vitro* experimental study, which may contribute to elucidate the mechanism underlying the action of NSAIDs on osteoblasts and therefore on bone.

## Material and Methods

### Osteoblast isolation and culture

**Figure 1:**
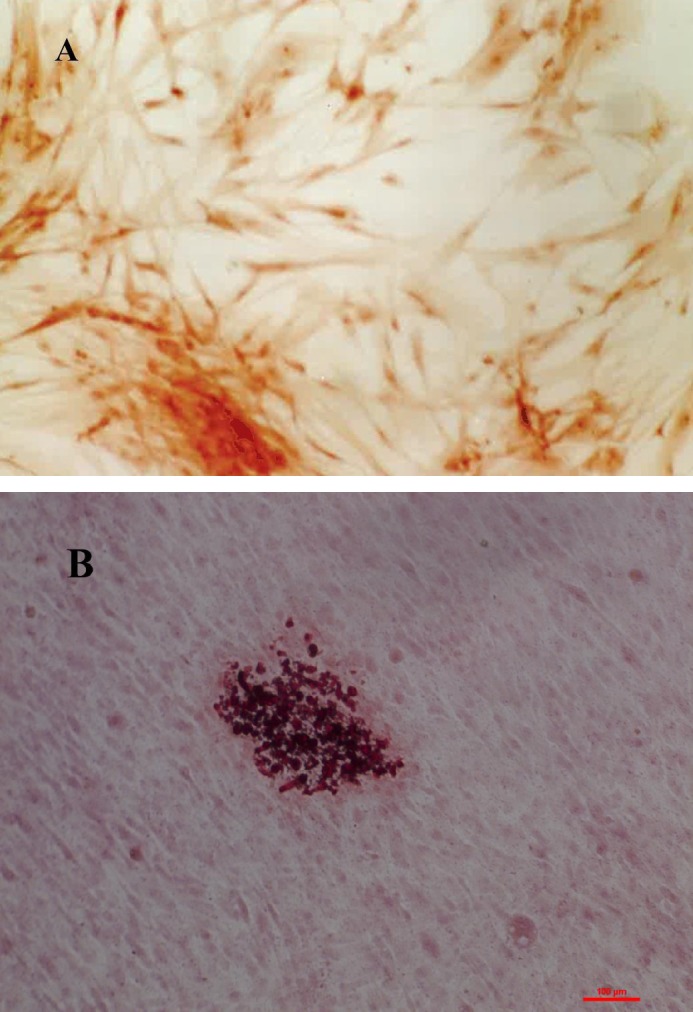
Characterization of the cell lines was made based on alkaline phosphatase activity, cells dyed in orange are positive for alkaline phosphatase activity (A) and mineralization in osteogenic medium, where it can be seen calcium nodes dyed in red color (B).

Three cell lines of primary culture human osteoblasts were established by isolating, characterizing, and culturing osteoblasts from bone sections obtained (under signed informed consent) during mandibular surgery from three Caucasian patients (2 women and 1 man) aged between 20 and 30 yrs, following the procedure of Manzano-Moreno et al. (2013). This study was in accordance with the ethical standards of the ethical committee of the University of Granada (reference no. 721). The characterization of the cell lines was made based on alkaline phosphatase (Fig. 1A) and mineralization in osteogenic medium (Fig. 1B). We have analyzed the alkaline phosphatase activity following the indications of Kit Sigma alkaline phosphatase (Sigma, St Louis, MO, USA): Confluent cells of different lines established were fixed in citrate-acetone-formaldehyde solution at room temperature. Cells were exposed to naphthol AS-BI phosphate (Sigma, St Louis, MO, USA), which were used as the substrate for ALP activity. Hematoxylin staining was used to determine the proportion of positive cells. For mineralization we have followed the method described by Manzano-Moreno et al. (2013): cells from the lines established from the two sample types were seeded (5 ×10^4^ cells/ml/well) in a six-well plate (Falcon, Becton Dickinson Labware, St. Louis, MO, USA) and cultured in complete medium supplemented with 5 mM *β*-glycerophosphate and 0.05 mM ascorbic acid at 37 °C in a humidified atmosphere of 95% air and 5% CO2. The medium was replaced after 4 days and then every 3 days. We examined the matrix mineralization of each cell line after 7, 15 and 22 days of culture. Red alizarin staining was used to visualize the precipitated calcium incorporated into the cellular matrix. Wells were washed with 150 mM sodium chloride, fixed in cold 70% ethanol for 5 min and rinsed three times with distilled water. Wells were then incubated for 10 min with 1 ml of a 2% red alizarin solution buffered at pH 4 with sodium hydroxide, then rinsed five times with distilled water and finally washed with PBS to reduce non-specific staining. Precipitate calcium present in the extracellular collagen matrix was colored red, revealing the mineralization nodules, which were counted under light microscopy.

### Treatments

The osteoblast cell lines were treated for 24 h with acetaminophen, indomethacin, ketoprofen, diclofenac, ibuprofen, ketorolac, naproxen, or piroxicam (Sigma, St. Louis, MO, USA) at a dose of 10 µM, untreated cells served as controls. Indomethacin, ketoprofen, diclofenac and piroxicam were previously dissolved with dimethyl Sulfoxide (DMSO) and diluted with culture medium, with a final concentration of DMSO of 0.001%.

### Determination of the gene expression by real-time polymerase chain reaction (RT-PCR)

To determine the effect of NSAIDs and acetaminophen on the osteoblast gene expression we followed the methodology described by Manzano-Moreno et al. (2018). mRNA was extracted from the treated cells with a silicate gel technique in the Qiagen RNeasy extraction kit (Qiagen Inc., Hilden, Germany). RNA was reverse-transcribed to cDNA and amplified by PCR using the iScript™ cDNA Synthesis Kit (Bio-Rad laboratories, Hercules, CA). mRNA of *RUNX2, OSX, OSC, COL-I, BMP-2, BMP-7, TGF-β1, TGFβ-R1, TGFβ-R2*, and *TGFβ-R3* was detected with primers designed using NCBI-nucleotide library and Primer3-design as listed in Table 1. Final results were normalized as described Ragni et al. (2013).

Quantitative RT-PCR (q-RT-PCR) was performed using the SsoFast™ EvaGreen® Supermix Kit (Bio-Rad laboratories) in accordance with the manufacturer’s protocol.

### Statistical analysis

SPSS 22.0 (IBM, Chicago, IL, USA) was used for data analyses. mRNA levels were expressed as means ± SD. The Kolmogorov–Smirnov test was applied to evaluate the normality of variable distributions. ANOVA test and Bonferroni corrections were used for multiple comparisons. *p* < 0.05 was considered significant in all tests. Three cell lines of primary culture human osteoblasts were used for all experiments, and at least three experiments were performed for all assays.

**Table 1:** Primer sequences for the amplification of cDNA by real-time PCR.

**Gene**	**Sense primer**	**Antisense primer**	**Amplicon (bp)**
TGF-*β*1	5′-TGAACCGGCCTTTCCTGCTTCTCATG-3′	5′-GCGGAAGTCAATGTACAGCTGCCGC-3′	152
TGF-*β* R1	5′-ACTGGCAGCTGTCATTGCTGGACCAG-3′	5′-CTGAGCCAGAACCTGACGTTGTCATATCA-3′	201
TGF-*β* R2	5′-GGCTCAACCACCAGGGCATCCAGAT-3′	5′-CTCCCCGAGAGCCTGTCCAGATGCT-3′	139
TGF-*β* R3	5′-ACCGTGATGGGCATTGCGTTTGCA-3′	5′-GTGCTCTGCGTGCTGCCGATGCTGT-3′	173
RUNX-2	5′-TGGTTAATCTCCGCAGGTCAC-3′	5′-ACTGTGCTGAAGAGGCTGTTTG-3′	143
OSX	5′-TGCCTAGAAGCCCTGAGAAA-3′	5′-TTTAACTTGGGGCCTTGAGA-3′	205
BMP-2	5′-TCGAAATTCCCCGTGACCAG-3′	5′-CCACTTCCACCACGAATCCA-3′	142
BMP-7	5′-CTGGTCTTTGTCTGCAGTGG-3′	5′-GTACCCCTCAACAAGGCTTC-3′	202
COL-I	5′-AGAACTGGTACATCAGCAAG-3′	5′-GAGTTTACAGGAAGCAGACA-3′	471
OSC	5′-CCATGAGAGCCCTCACACTCC-3′	5′-GGTCAGCCAACTCGTCACAGTC-3′	258
UBC	5′-TGGGATGCAAATCTTCGTGAAGACCCTGAC-3′	5′-ACCAAGTGCAGAGTGGACTCTTTCTGGATG-3′	213
PPIA	5′-CCATGGCAAATGCTGGACCCAACACAAATG-3′	5′-TCCTGAGCTACAGAAGGAATGATCTGGTGG-3′	256
RPS13	5′-GGTGTTGCACAAGTACGTTTTGTGACAGGC-3′	5′-TCATATTTCCAATTGGGAGGGAGGACTCGC-3′	251

## Results

### Effect of NSAIDs on the expression of *RUNX-2, COL-I, OSX,* and *OSC* genes

**Figure 2:**
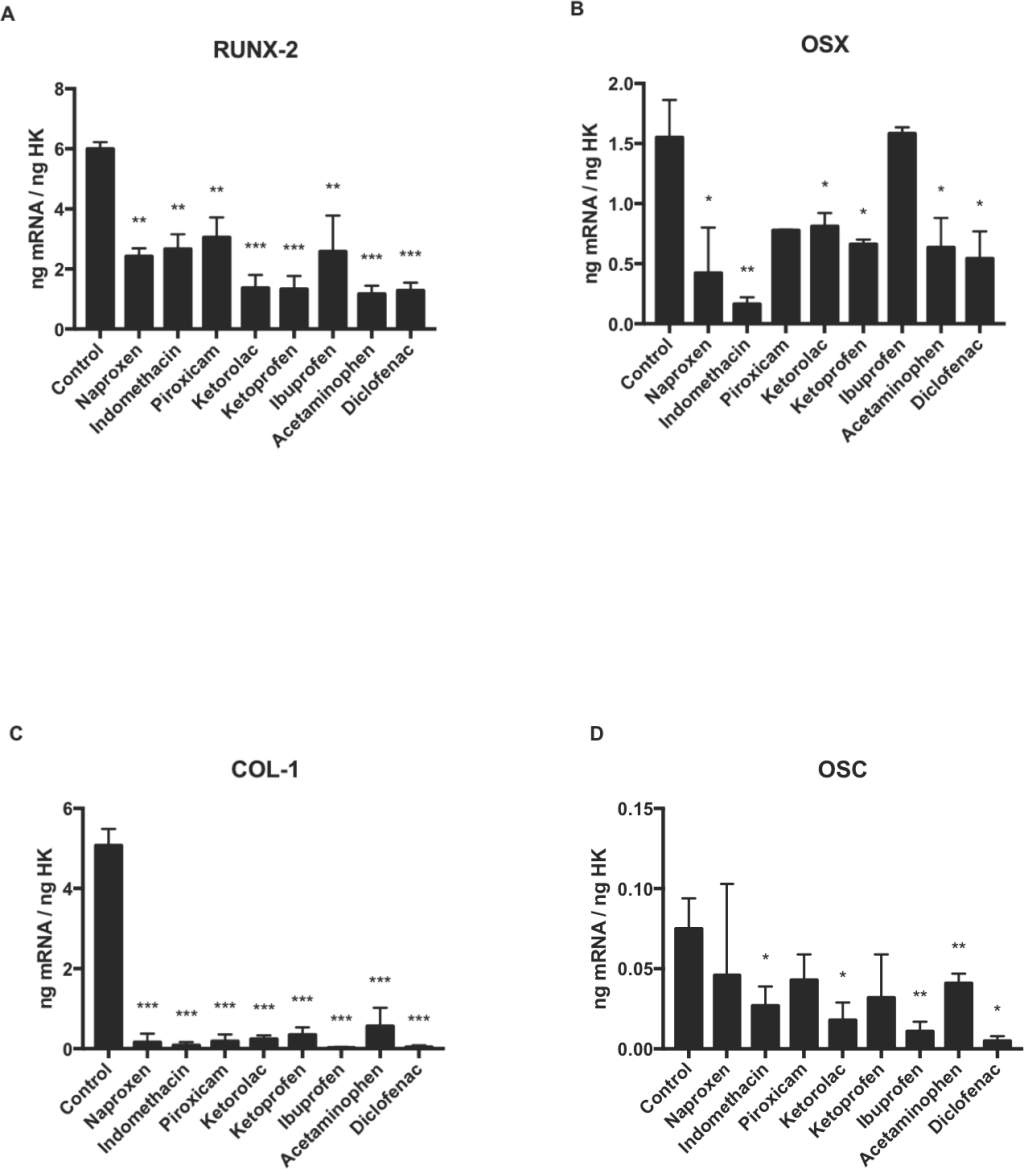
Expression of osteoblast differentiation genes treated for 24 h with acetaminophen, indomethacin, ketoprofen, diclofenac, ibuprofen, ketorolac, naproxen, or piroxicam (10 µM). (A) RUNX-2, (B) OSX, (C) COL-I, (D) OSC. Data are expressed as means ± SD of ng of mRNA per average ng of housekeeping mRNAs. *p ≤ 0.047, **p ≤ 0.007, ***p ≤ 0.0001.

Quantitative RT-PCR (q-RT-PCR) analysis was used to evaluate the expression of the osteoblast differentiation makers, *RUNX-2* (Fig. 2A), *OSX* (Fig. 2B), *COL-I* (Fig. 2C), and *OSC* (Fig. 2D). All genes expression decreased after 24 h of osteoblast treatment with each studied drugs except for *OSX* (Fig. 1B) which did not change with ibuprofen, and *OSC* (Fig. 2D), whose expression was decreased after treatment with acetaminophen, indomethacin, diclofenac, ibuprofen, or ketorolac but it did not change after treatment with ketoprofen, naproxen or piroxicam.

### Effect of NSAIDs on gene expression of *BMP-2* and *BMP-7*

**Figure 3:**
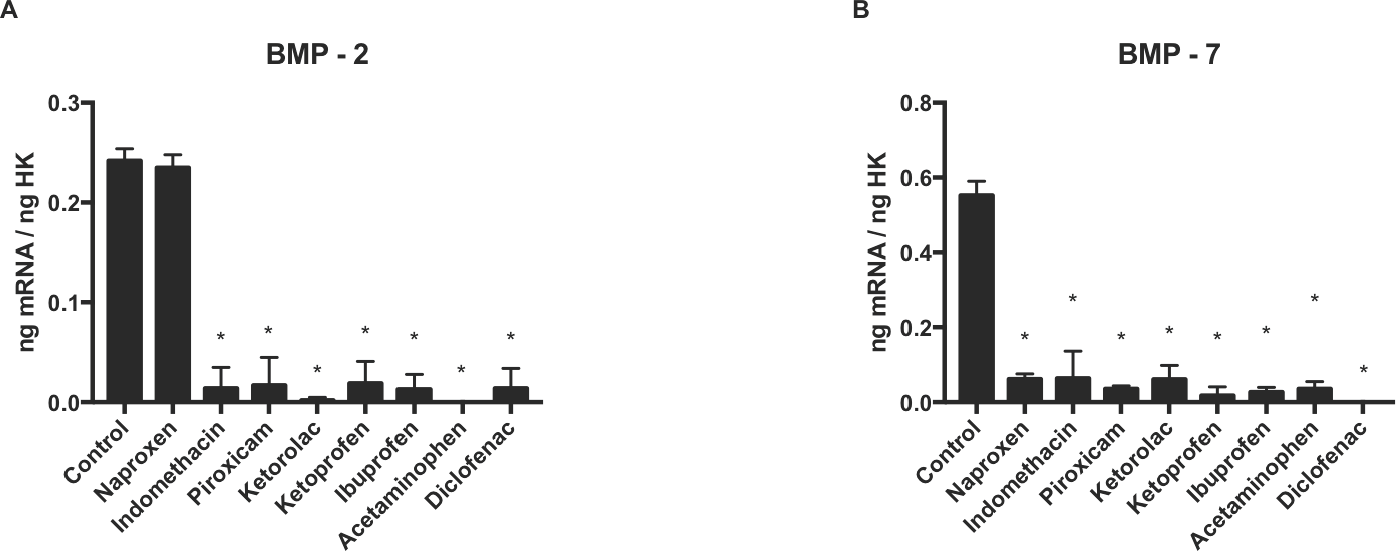
Expression of osteoblast genes treated for 24 h with acetaminophen, indomethacin, ketoprofen, diclofenac, ibuprofen, ketorolac, naproxen, or piroxicam (10 µM). (A) BMP-2, (B) BMP-7. Data are expressed as ng of mRNA per average means ± SD of ng of housekeeping mRNAs. *p ≤ 0.001.

Figure 2 and Table 1 depicts q-RT-PCR results for expression of the growth factors *BMP-2* and *BMP-7*. After 24 h of treatment at a dose of 10 µM, osteoblast expression of *BMP-2* (Fig. 3A) and *BMP-7* (Fig. 3B) was significantly lower versus controls with all drugs assayed except for naproxen, which did not modify *BMP-2* expression.

### Effect of NSAIDs on the gene expression of ***TFG-β1*and its receptors (*TFGβ-R1, TFGβ-R2, and TFGβ-R3*)**

**Figure 4:**
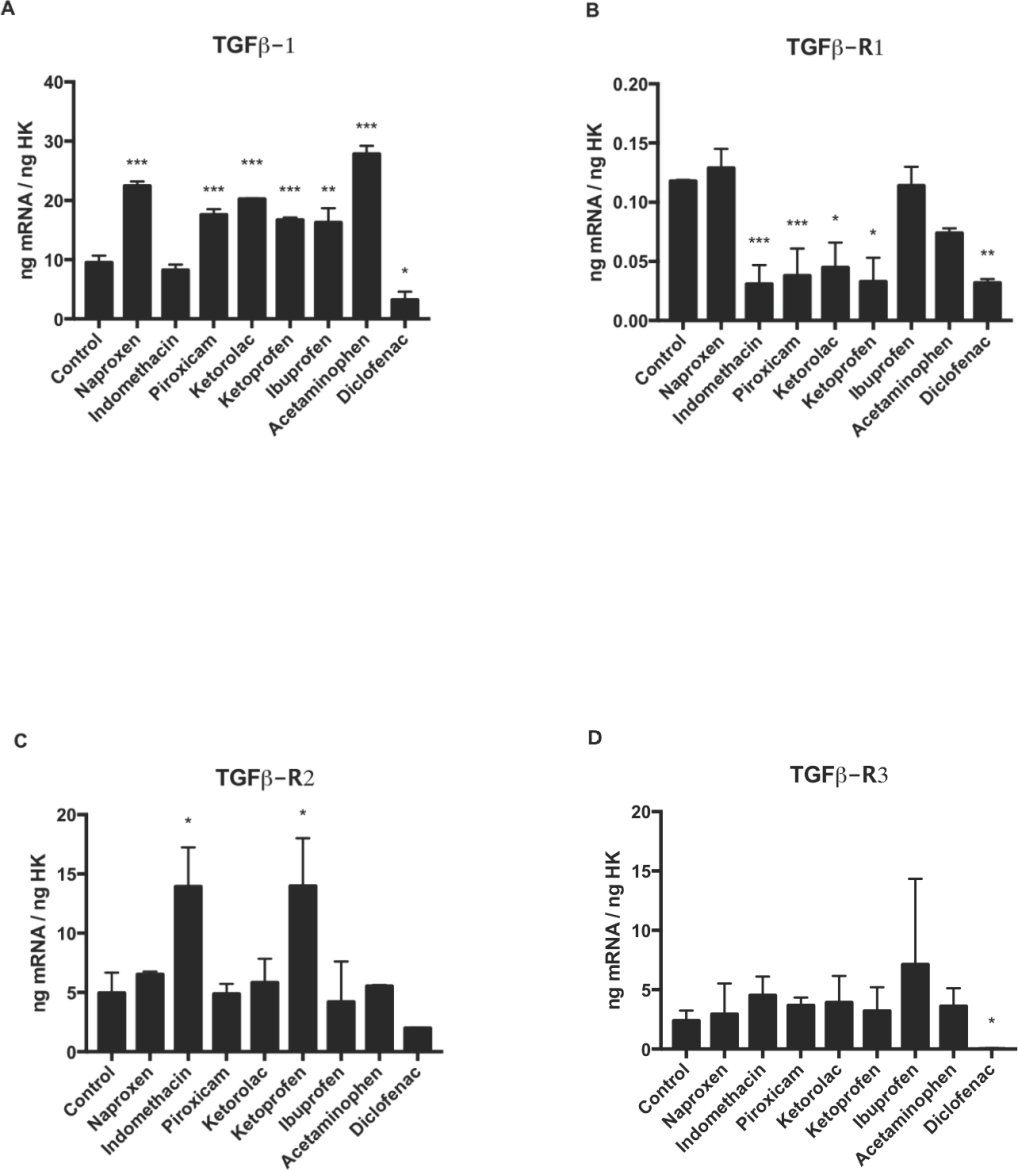
Expression of osteoblast genes treated for 24 h with acetaminophen, indomethacin, ketoprofen, diclofenac, ibuprofen, ketorolac, naproxen, or piroxicam (10 µM). (A) TFG-*β*1, (B) TFG*β*R1, (C) TFG*β*R2, (D) TFG*β*R3. Data are expressed as means ± SD of ng of mRNA per average ng of housekeeping mRNAs. *p ≤ 0.032, **p ≤ 0.006, ***p ≤ 0.001.

Figure 3 depicts q-RT-PCR results for the gene expression of *TGF-β1* and its receptors (*TGFβR1, TGFβR2, and TGFβR3*). After 24 h of treatment at a dose of 10 µM, osteoblast expression of *TGF-β1* (Fig. 4A) expression was higher versus controls with all drugs assayed except for diclofenac, which produced a reduction in this expression and indomethacin that unchanged the expression. *TGFβR1* (Fig. 4B) expression was decreased versus controls after treatment with each drug except for ibuprofen and naproxen, which did not affect this expression. *TGFβR2* (Fig. 4C) expression was not changed by treatment with any NSAID except for indomethacin and ketoprofen, which significantly increased this expression. *TGFβR3* (Fig. 4D) was also unchanged by treatment with any NSAID except for diclofenac, which significantly reduced this expression.

## Discussion

The results of this *in vitro* study of three osteoblast cell lines demonstrate that the expression of genes involved in osteoblast growth, maturation, and function can be modulated by treatment with acetaminophen, indomethacin, ketoprofen, diclofenac, ibuprofen, ketorolac, naproxen, and piroxicam at a dose in the therapeutic range (Chang et al., 2009; Buckley & Brogden, 1990). The dosage tested (10 µM) was selected based on previous studies which showed that this therapeutic dose exerts an effect in osteoblast physiology without producing any kind of cytotoxicity (necrosis) on this cell population (Chang et al., 2009; De Luna-Bertos et al., 2015). Paracetamol is widely administered in clinical practice for its analgesic and antipyretic properties. It is not currently considered to be in the group of NSAIDs but was included in our study because of its capacity for non-competitive reversible inhibition of the cyclooxygenase enzyme (Dawson et al., 2005).

Most of these drugs inhibited the gene expression of *BMP-2* and *BMP-7*, which are important molecules for osteoblast growth and differentiation, while they increased the expression of *TGF-β1* but not its receptors and reduced the expression of *RUNX-2*, *COL-I*, *OSX* and *OSC*, which are directly related to cell maturation. These data contribute to completing knowledge on the effect of NSAIDs on molecular, cellular, and functional parameters of osteoblasts (García-Martínez et al., 2015; De Luna-Bertos et al., 2015; De Luna-Bertos et al., 2013) and further elucidate the mechanisms that underlie the effects of NSAIDs on these bone-forming cells.

Osteoprogenitors from the medulla differentiate and mature into pre-osteoblasts, osteoblasts, and osteocytes. Each stage of the functional differentiation of osteoblasts (proliferation, bone matrix synthesis, and mineralization) has been associated with specific cell markers (Long, 2011). In the present *in vitro* assays, markers related to each stage were modulated by NSAID treatment, suggesting changes in the differentiation and/or maturation of osteoblasts and therefore in their function.

The expression of *RUNX-2*, *OSX*, *COL-I*, and *OSC* genes was reduced in the human osteoblastic cell lines after treatment with all eight NSAIDs. Except *OSX* and *OSC* that did not change with ibuprofen and with ketoprofen, naproxen, or piroxicam, respectively. *RUNX-2* and *OSX* expressions are essential for osteoblast differentiation, with *RUNX-2* being more closely related to proliferation and OSX to the final maturation stage (Capulli, Paone & Rucci, 2014). *RUNX-2* is also involved in the expression of other genes related to osteoblast maturation, including *COL-I,* alkaline phosphatase (*ALP*), and *OSC* (Fakhry et al., 2013). *COL-I* is associated with the proliferative stage, *ALP* with the differentiation stage, and *OSC* with the final maturation stage, which is characterized by the increased expression of *OSX* and *OSC* genes (Nakashima et al., 2002; Glass et al., 2005; Hu et al., 2005). It should be borne in mind that the signaling relays in each stage are responsible for the final gene expression.

These observations of the inhibitory effects of NSAIDs on osteoblast differentiation and maturation are consistent with reports on the reduction in *ALP* or *OSC* synthesis and extracellular matrix mineralization in NSAID-treated osteoblastic cells (Díaz-Rodríguez et al., 2010; De Luna-Bertos et al., 2015; Díaz-Rodríguez et al., 2012a; Díaz-Rodríguez et al., 2012b; Arpornmaeklong, Akarawatcharangura & Pripatnanont, 2008).

*TGF-β1* and *BMP* signaling has a critical regulatory function in osteoblast differentiation and bone formation (Chen, Deng & Li, 2012; Rahman et al., 2015), while members of the *BMP* family are also involved in regulating osteoblast lineage-specific differentiation and subsequent bone formation, inducing bone formation and being expressed during bone repair. *BMP-2* and *BMP-7* play a key role in osteoblast differentiation (Beederman et al., 2013) and their involvement in bone formation has led to their clinical application (Bayat et al., 2015; Seo et al., 2015; Kelly, Vaughn & Anderson, 2016; Lin et al., 2016). A major inhibition of *BMP-2* and *BMP-7* expression, implying the arrest of differentiation, was observed after treatment with all of the studied NSAIDs except for naproxen. In contrast, all of them except for diclofenac increased the expression of *TGF-β1* but not of its receptors, whose expression was either reduced or unchanged by the treatment, probably affecting the action of *TGF-β1*.

We have to highlight that results from this work has been obtained from three different cell lines, which would suppose a limitation.

## Conclusions

According to the present *in vitro* study, NSAIDs and acetaminophen can modulate the expression of genes directly involved in osteoblast physiology, suggesting an inhibition of the maturation process that would directly affect bone tissue. Given the potential clinical repercussions of these findings, *in vivo* studies are warranted to verify and further explore the relationships found.

##  Supplemental Information

10.7717/peerj.5415/supp-1 Data S1 Raw data

## Additional Information and Declarations

### Data Availability

The following information was supplied regarding data availability: The raw data are provided in Supplemental File.
