# Supplementary material for: Repercussion of nonsteroidal anti-inflammatory drugs on the gene expression of human osteoblasts
Source: PeerJ. 2018 Aug 14;6:e5415. doi: 10.7717/peerj.5415 (PMC6097469; doi:10.7717/peerj.5415)
Supplement: Data S1 [file peerj-06-5415-s001.docx]

|  | **TREATMENT** | **Mean ± SD** | ***P*** |
| --- | --- | --- | --- |
| BMP-2 | control | 0,242 ± 0,012 | - |
|  | Naproxeno | 0,235 ± 0,013 | 1,000 |
|  | Indomethacin | 0,014 ± 0,021 | **0,000** |
|  | Piroxicam | 0,017 ± 0,028 | **0,000** |
|  | Ketorolac | 0,002 ± 0,003 | **0,000** |
|  | Ketoprofen | 0,019 ± 0,022 | **0,000** |
|  | Ibuprofen | 0,013 ± 0,015 | **0,000** |
|  | Acetaminophen | 0,000 ± 0,000 | **0,000** |
|  | Diclofenac | 0,014 ± 0,02 | **0,000** |
| BMP-7 | control | 0,552 ± 0,038 | - |
|  | Naproxeno | 0,062 ± 0,014 | **0,000** |
|  | Indomethacin | 0,064 ± 0,072 | **0,000** |
|  | Piroxicam | 0,036 ± 0,008 | **0,000** |
|  | Ketorolac | 0,061 ± 0,037 | **0,000** |
|  | Ketoprofen | 0,018 ± 0,023 | **0,000** |
|  | Ibuprofen | 0,027 ± 0,013 | **0,000** |
|  | Acetaminophen | 0,036 ±0,019 | **0,000** |
|  | Diclofenac | 0,0005 ± 0,0004 | **0,000** |
| TGFβ-1 | control | 9,519 ± 1,17 | - |
|  | Naproxeno | 22,462 ± 0,705 | **0,001** |
|  | Indomethacin | 8,253 ± 0,931 | 0,295 |
|  | Piroxicam | 17,593 ± 0,922 | **0,001** |
|  | Ketorolac | 20,244 ± 0,076 | **0,001** |
|  | Ketoprofen | 16,726 ± 0,394 | **0,001** |
|  | Ibuprofen | 16,273 ± 2,418 | **0,006** |
|  | Acetaminophen | 27,836 ± 1,396 | **0,001** |
|  | Diclofenac | 3,229 ± 1,38 | **0,012** |
| TGFβ-R1 | control | 0,118 ± 0,001 | - |
|  | Naproxeno | 0,129 ± 0,016 | 1,000 |
|  | Indomethacin | 0,031 ± 0,016 | **0,001** |
|  | Piroxicam | 0,038 ± 0,023 | **0,001** |
|  | Ketorolac | 0,045 ± 0,021 | **0,010** |
|  | Ketoprofen | 0,033 ± 0,02 | **0,018** |
|  | Ibuprofen | 0,114 ± 0,016 | 1,000 |
|  | Acetaminophen | 0,074 ± 0,004 | **0,045** |
|  | Diclofenac | 0,032 ± 0,003 | **0,002** |
| TGFβ-R2 | control | 4,958 ± 1,719 | - |
|  | Naproxeno | 6,528 ± 0,238 | 1,000 |
|  | Indomethacin | 13,924 ± 3,313 | **0,032** |
|  | Piroxicam | 4,877 ± 0,854 | 1,000 |
|  | Ketorolac | 5,838 ± 2,002 | 1,000 |
|  | Ketoprofen | 13,974 ± 4,034 | **0,032** |
|  | Ibuprofen | 4,203 ± 3,409 | 1,000 |
|  | Acetaminophen | 5,518 ± 0,114 | 1,000 |
|  | Diclofenac | 1,987 ± 0,023 | 1,000 |
| TGFβ-R3 | control | 2,392 ± 0,845 | - |
|  | Naproxeno | 2,949 ± 2,57 | 0,812 |
|  | Indomethacin | 4,527 ± 1,583 | 0,130 |
|  | Piroxicam | 3,669 ± 0,672 | 0,110 |
|  | Ketorolac | 3,933 ± 2,231 | 0,501 |
|  | Ketoprofen | 3,206 ± 1,997 | 0,551 |
|  | Ibuprofen | 7,127 ± 7,209 | 0,373 |
|  | Acetaminophen | 3,616 ± 1,512 | 0,315 |
|  | Diclofenac | 0,029 ± 0,04 | **0,033** |
| RUNX-2 | control | 6,000 ± 0,226 | - |
|  | Naproxeno | 2,425 ± 0,264 | **0,001** |
|  | Indomethacin | 2,660 ± 0,498 | **0,001** |
|  | Piroxicam | 3,049 ± 0,668 | **0,002** |
|  | Ketorolac | 1,371 ± 0,431 | **0,000** |
|  | Ketoprofen | 1,327 ± 0,445 | **0,000** |
|  | Ibuprofen | 2,580 ± 1,194 | **0,001** |
|  | Acetaminophen | 1,169 ± 0,276 | **0,000** |
|  | Diclofenac | 1,286 ± 0,256 | **0,000** |
| OSX | control | 1,551 ± 0,312 | - |
|  | Naproxeno | 0,423 ± 0,378 | **0,008** |
|  | Indomethacin | 0,164 ± 0,057 | **0,001** |
|  | Piroxicam | 0,777 ± 0,005 | **0,045** |
|  | Ketorolac | 0,810 ± 0,112 | **0,037** |
|  | Ketoprofen | 0,663 ± 0,037 | **0,032** |
|  | Ibuprofen | 1,583 ± 0,052 | 1,000 |
|  | Acetaminophen | 0,635 ± 0,246 | **0,037** |
|  | Diclofenac | 0,543 ± 0,226 | **0,019** |
| COL-1 | control | 5,070 ± 0,415 | - |
|  | Naproxeno | 0,164 ± 0,212 | **0,000** |
|  | Indomethacin | 0,088 ± 0,079 | **0,000** |
|  | Piroxicam | 0,187 ± 0,171 | **0,000** |
|  | Ketorolac | 0,244 ± 0,088 | **0,000** |
|  | Ketoprofen | 0,348 ± 0,184 | **0,000** |
|  | Ibuprofen | 0,021 ± 0,022 | **0,000** |
|  | Acetaminophen | 0,563 ± 0,461 | **0,000** |
|  | Diclofenac | 0,036 ± 0,05 | **0,000** |
| OSC | control | 0,075 ± 0,019 | - |
|  | Naproxeno | 0,046 ± 0,057 | 1,000 |
|  | Indomethacin | 0,027 ± 0,012 | **0,025** |
|  | Piroxicam | 0,043 ± 0,016 | 0,092 |
|  | Ketorolac | 0,018 ± 0,011 | **0,029** |
|  | Ketoprofen | 0,032 ± 0,027 | 1,000 |
|  | Ibuprofen | 0,011 ± 0,006 | **0,004** |
|  | Acetaminophen | 0,041 ± 0,006 | **0,007** |
|  | Diclofenac | 0,286 ± 0,138 | **0,047** |
